# StakeMeter: Value-Based Stakeholder Identification and Quantification Framework for Value-Based Software Systems

**DOI:** 10.1371/journal.pone.0121344

**Published:** 2015-03-23

**Authors:** Muhammad Imran Babar, Masitah Ghazali, Dayang N. A. Jawawi, Kashif Bin Zaheer

**Affiliations:** 1 Department of Software Engineering, Universiti Teknologi Malaysia, Johor, Malaysia; 2 Department of Mathematical Sciences, Universiti Teknologi Malaysia, Johor, Malaysia; Tianjin University of Technology, CHINA

## Abstract

Value-based requirements engineering plays a vital role in the development of value-based software (VBS). Stakeholders are the key players in the requirements engineering process, and the selection of critical stakeholders for the VBS systems is highly desirable. Based on the stakeholder requirements, the innovative or value-based idea is realized. The quality of the VBS system is associated with the concrete set of valuable requirements, and the valuable requirements can only be obtained if all the relevant valuable stakeholders participate in the requirements elicitation phase. The existing value-based approaches focus on the design of the VBS systems. However, the focus on the valuable stakeholders and requirements is inadequate. The current stakeholder identification and quantification (SIQ) approaches are neither state-of-the-art nor systematic for the VBS systems. The existing approaches are time-consuming, complex and inconsistent which makes the initiation process difficult. Moreover, the main motivation of this research is that the existing SIQ approaches do not provide the low level implementation details for SIQ initiation and stakeholder metrics for quantification. Hence, keeping in view the existing SIQ problems, this research contributes in the form of a new SIQ framework called ‘*StakeMeter*’. The *StakeMeter* framework is verified and validated through case studies. The proposed framework provides low-level implementation guidelines, attributes, metrics, quantification criteria and application procedure as compared to the other methods. The proposed framework solves the issues of stakeholder quantification or prioritization, higher time consumption, complexity, and process initiation. The framework helps in the selection of highly critical stakeholders for the VBS systems with less judgmental error.

## Introduction

In the Requirements Engineering (RE), the functional and non-functional goals are documented and analyzed in order to develop a new system [1, 2]{Wiegers, 1999 #64}. The well documented and right requirements have a prominent effect on the quality of the software [3, 4]. The RE process is a combination of different activities or phases which when performed in collaboration results in a requirements document. The different phases of the RE process are elicitation, analysis, specification, validation and management [5, 6].

**Fig 1:**
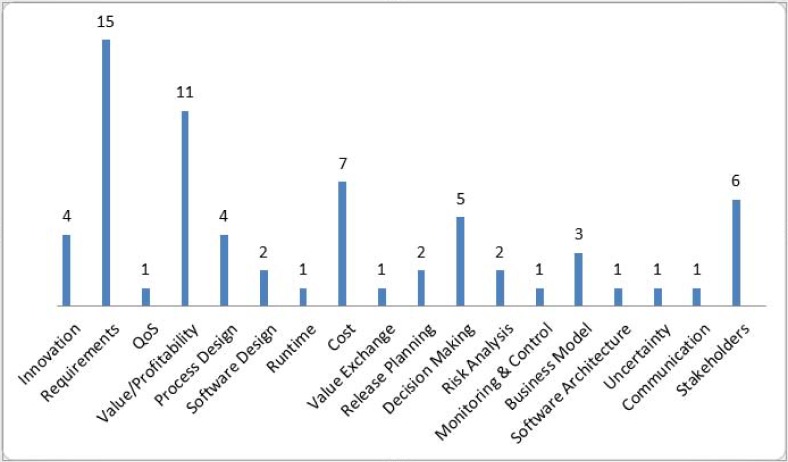
Issues highlighted in the VBS Research.

The VBS systems are the part of value-based software engineering (VBSE). The definition of VBSE given by Barry Boehm is “the explicit concern with value concerns in the application of science and mathematics by which properties of computer software are made useful to the people” [7]. Hence, a VBS system is one that provides useful software properties to the intended stakeholders. The VBS systems are based on an innovative or value-based idea and are associated with the economic leverage. The implementation of an innovative or value-based idea is difficult in terms of high uncertainty. It is difficult to guess that either the innovative or value-based idea will yield the economic benefit or not. In the VBS systems, the involvement of relevant stakeholders can play a vital role in the quality improvement [8] and value-based software requirements engineering (VBSRE) practices. The case of the VBS systems is very sensitive because they are usually part of the distributed environments due to the diverse locations of the stakeholders. In such cases, it is very difficult to consider all the stakeholders who have little stakes in the development of the VBS system. (Fig. 1) summarizes the research on the VBS systems and shows the frequency of the research conducted on different issues in order to design high-quality VBS systems. The research in the domain of VBS development mainly focuses on requirements, profitability, cost and decision-making. The VBSRE approaches do not focus on the stakeholder analysis. There are few studies that focus on the significance of the stakeholders for the VBS systems, but do not propose any framework or approach for the stakeholder identification and quantification (SIQ) process. There is the need to integrate the stakeholder analysis with the VBSRE for better RE practices.

Stakeholders are the key element in the success and failure of the software. Hence, the high priority is given to the stakeholders in the Star Triangle in [9]. A comprehensive analysis of the SIQ problems is discussed in [10] for the VBS development and it is forced that “to characterise the stakeholders based on the responsibilities”, “to propose the stakeholders’ metrics to make the SIQ process easy” and “to propose an SIQ framework in easy steps” for the VBS systems. Freeman defines a stakeholder as “any group or individual who can affect or is affected by the achievement of the organization’s objectives” [11]. Tom Gilb defines a stakeholder as “any person or organizational group with an interest in, or ability to affect, the system or its environment”[12]. The stakeholder or customer satisfaction related to the VBS system mainly depends on the selection of highly critical requirements. In order to satisfy the customers, the selection of highly valuable stakeholders is essential in the requirements elicitation phase (REP). In order to gain the market leverage, the trustworthiness of the system must be high. The VBS trustworthiness or reliability is taken as a quality measure as trustworthiness or reliability of cloud service is considered as a quantitative quality measure in the case of cloud computing [13]. The cloud computing is also a part of the value-based paradigm and the trustworthiness of the cloud services is highly desirable. Same is the case with the stakeholders with respect to the trustworthiness. However, the current SIQ techniques cannot be adopted as a standard because they are not standardized, applied and tested in a real time environment. Hence, the suitability of the existing SIQ techniques for the VBS systems is questionable. The VBS systems are associated with the economic leverage, thus all the entities cannot be taken at par. For the VBS systems, only the key stakeholders are considered during the requirements analysis phase. It is not easy to decide which technique is suitable for the VBS development. The selection of a technique as a model is very difficult because some methods are just characterizing the stakeholders instead of the quantification [14, 15]. Enough work is done with respect to the value-based requirements prioritization or quantification in different research studies like [16–19]. However, the work in the domain of value-based stakeholder prioritization or quantification is not sufficient.

The rest of the paper is divided into 9 sections. Section 2 discusses the SIQ research background. The detail of the proposed SIQ framework *StakeMeter* is given in Section 3. Section 4 is about stakeholder factor formulation. The description of inclusion and exclusion criteria of stakeholders is given in Section 5. Section 6 describes the implementation guidelines. The details of the case studies are given in Section 7. The details of performance analysis of the *StakeMeter* are given in Section 8. Section 9 highlights the future research directions. The last section, Section 10 concludes this study.

## Research Background

Different techniques and methodologies are presented to identify and quantify the stakeholders. Currently there is a lack of uniformity in the existing techniques. There is no existence of a uniform SIQ framework and the selection of the existing approaches is complex [14, 15]. The current methods and techniques do not provide clear guidelines, thus the identification and selection of valuable stakeholders is very difficult. The current techniques identify the stakeholders based on their relationships, roles and influence [20–23]. They provide a very high level picture of the business entities instead of focusing on low level details. There are some techniques that do not consider the aspects of relationships, roles and influence [24, 25] which shows the non-uniformity of the existing techniques. The attributes of power, legitimacy, and urgency are taken into account in the Mitchell’s theory. These attributes are used to divide the stakeholders into eight categories like dormant stakeholders, discretionary stakeholders, demanding stakeholders, dominant stakeholders, dependent stakeholders, dangerous stakeholders, definitive stakeholders and non-stakeholders [21]. Mitchell’s model is a very basic initial model for stakeholders’ identification and lacks in low level details.

Ballejos and Montagna presented a technique based on the roles and types for the SIQ at inter-organizational level [26–28]. The stakeholders are quantified based on key attributes of function, knowledge abilities, geographical position and hierarchy level. The technique induces the problem of competency measurement among all four aspects. Moreover, the technique is not cost-effective in terms of time utilization.

The PisoSIA (Stakeholder Identification and Analysis) technique is an extension in an existing technique called PISO (Process Improvement for Strategic Objectives) [29]. The technique does not focus on the SIQ process. The technique focuses on the identification of new stakeholders when a change is required in the existing functionality of a system. However, the importance of the stakeholders is not denied. Boonstra (2006) conducted a research called “ERP-implementation project from a stakeholder perspective”. In this research, a technique is presented based on the Mitchell’s model to classify the stakeholders at a higher level of abstraction [30]. In this technique, the new stakeholders are identified based on the induced change and the impact of the change is measured on the existing stakeholders.

Glinz and Wieringa quantified stakeholders into three major categories i.e. critical, major and minor [31]. However, the process level details are not given for the SIQ. An abstract picture of the identification and classification model is depicted. Thus, it is difficult to adopt the model when a project or product requires an agile environment in terms of its execution, implementation or development. Woolridge *et al*. (2007) divide stakeholders into high level major categories based on their induced risk. The research does not provide in depth process level details for classification of stakeholders. The reported stakeholder categories are financial supporters, customers, internal stakeholders, external stakeholders, special interest stakeholders and influencer stakeholders [32].

**Fig 2:**
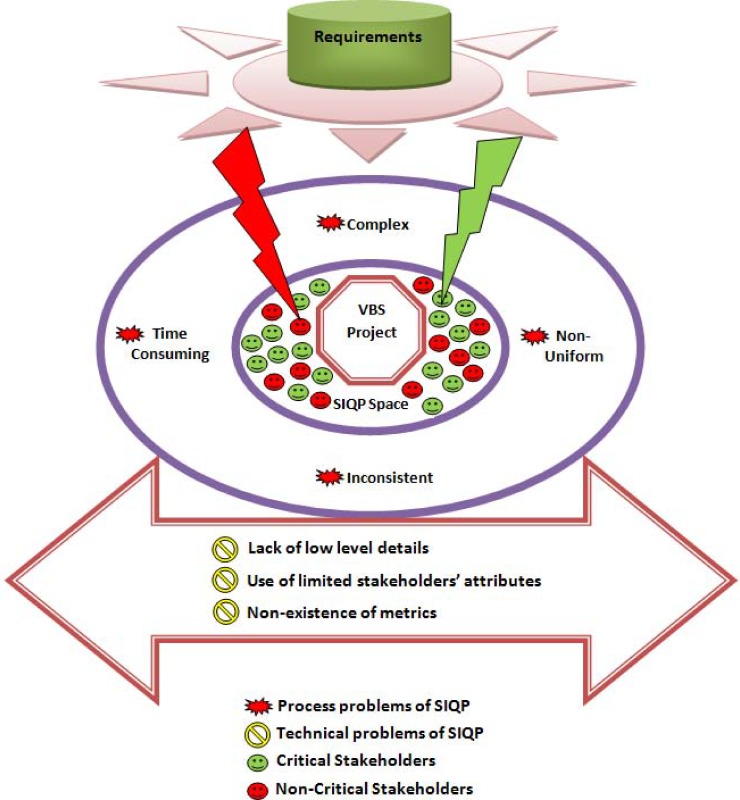
Problems of the SIQP.

The research motivation is based on the findings which state that the current techniques are complex, provide a description of stakeholders at a higher level of abstraction, and do not provide process level details in order to quantify the stakeholders [29], incorrect early findings [29], are not uniform [33], cannot be adopted as a framework [33], and are time consuming and costly [26–28]. The VBS systems mainly focus on the economic leverage and this thing differentiates the VBS from traditional software applications. There is the need to propose a new SIQ framework for the VBS systems. The proposed framework will help in finding out a critical set of stakeholders and decision making. The framework provides clear and easy guidelines to initiate the SIQ process. The concept of multi-attribute and multi-metrics as proposed in [34, 35] is introduced in order to evaluate the trustworthiness or reliability of the stakeholders. The selected critical stakeholders will help the requirement engineers in the elicitation of precise and accurate requirements in terms of system’s functionality and usability. The clear and profound requirements will help the developers to develop a useful system of high quality as per needs of the stakeholders. (Fig. 2) describes the different issues of the SIQ. There are two types of problems named as process problems and technical problems of the SIQ. The green lightning sign in (Fig. 2) indicates the requirements gathered from the success critical stakeholders while the red lightning sign indicates the requirements which may cause a high risk. In order to solve the SIQ problems this research contributes in the form of a new SIQ framework called as *StakeMeter*. The proposed framework *StakeMeter* comprises responsibilities of stakeholders, attributes and metrics as key contribution elements.

## Proposed Framework StakeMeter

**Fig 3:**
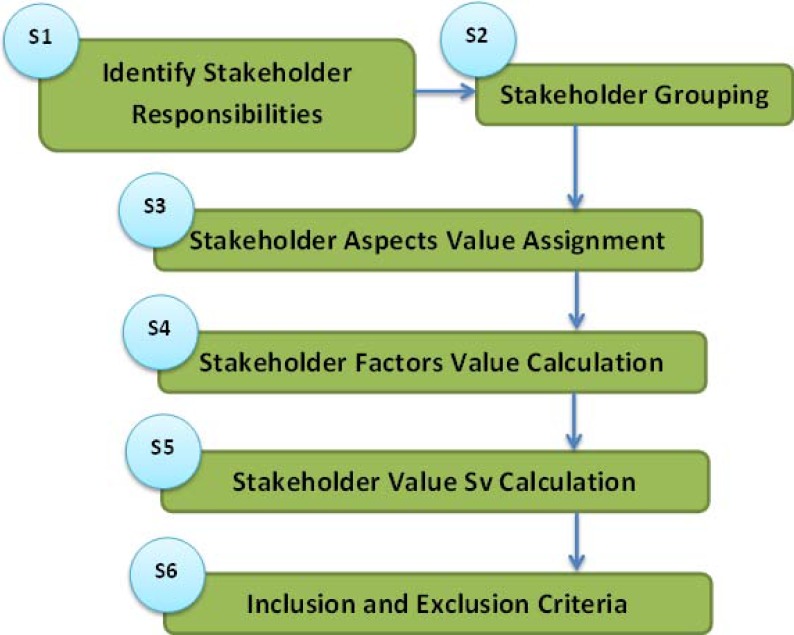
Flowchart of StakeMeter.

The proposed SIQ framework called *StakeMeter* comprises 6 steps. The main steps of the framework are stakeholder responsibilities, stakeholder groups, stakeholder aspects or attributes, stakeholder factors, stakeholder values and inclusion and exclusion criteria. (Fig. 3) shows the different steps of the proposed framework *StakeMeter*.

### 3.1 Stakeholder Responsibilities

The organizations mostly define the responsibilities of their employees from higher level to the lower level including their authorities. The overview of such documents may help in understanding the responsibilities of the stakeholders of an organization. If the responsibility documents are not available, then there is the need to create them. The responsibilities of stakeholders play a vital role in identification of different stakeholders who may contribute in the development of the VBS systems.

### 3.2 Stakeholder Groups

In this step, the stakeholders are identified and divided into different groups based on their responsibilities. Stakeholders or people working in the same vicinity and professional area are placed in the same group. Moreover, based on the job relationships the stakeholders may also be placed in the similar groups. However, their level of professionalism is defined using stakeholder factors or metrics which are described in Section 4.

### 3.3 Stakeholder Aspects or Attributes

An initial estimation of all the factors is based on the stakeholder aspects or attributes which are taken into account during quantification of the stakeholders. These aspects are taken from literature and industry professionals. Some of the stakeholder aspects are communication, interpretation, decision making, cognitive load, complexity, clarity, objectivity, self-confidence, language barriers, time and geographical differences. The description of some of the stakeholder aspects is given in Table 1. However, the selected stakeholder aspects are described in detail in Section 4.

**Table 1:** Stakeholders’ aspects.

Aspect Name	Aspect Description
Communication	The stakeholders’ ability to communicate properly.
Interpretation	Stakeholders’ ability to describe the economic benefit of the required needs.
Decision Making	The stakeholder has a prominent role in decision making or not.
Cognitive Load	This shows the stakeholder’s ability related to memory stress.
Complexity	Stakeholder’s ability to present the complex needs in an elaborative way.
Clarity	Stakeholder’s ability to describe the intended needs In a clear manner.
Objectivity	Stakeholder’s ability to describe the intended meanings of the needs properly.
Self Confidence	It represents the level of self-confidence of the stakeholder.

### 3.4 Stakeholder Factors

In this step, the individual value of each of the stakeholder factor or metric is calculated to find out the value of a stakeholder. These individual values are given as an input to the *Sv* function as shown in Section 4, and the final value of the stakeholder *Sv* is calculated. This step of the framework comprises different stakeholder factors that must be considered during the SIQ to select the most critical stakeholders. The value of these factors is calculated to find out the importance of an entity for a given VBS system. Table 2 describes the proposed factors in detail.

**Table 2:** Factor description.

Factor Name	Acronym	Factor Description
Risk Factor	F_SR_	Depicts the risk imposed by a stakeholder.
Instability Factor	F_SI_	Helps in calculating the instability in stakeholder’s nature.
Communication Factor	F_SC_	Vital in finding out stakeholder’s fluency about the ideas.
Skill Factor	F_SS_	Helps in finding out stakeholders’ professional abilities.
Interest Factor	F_SIT_	Helpful in knowing the stakeholder’s interests in system.
Personality Factor	F_SP_	Helps in observing the personality of a stakeholder.
Hierarchy Factor	F_SH_	The F_SH_ helps to find out that at which extent the level of hierarchy is dominant on the personality of a stakeholder.
Legitimacy Factor	F_SLG_	Depicts the stakeholder have some legitimate need or not.
Environment Factor	F_SE_	Helpful in finding out the professional ethics.

**Fig 4:**
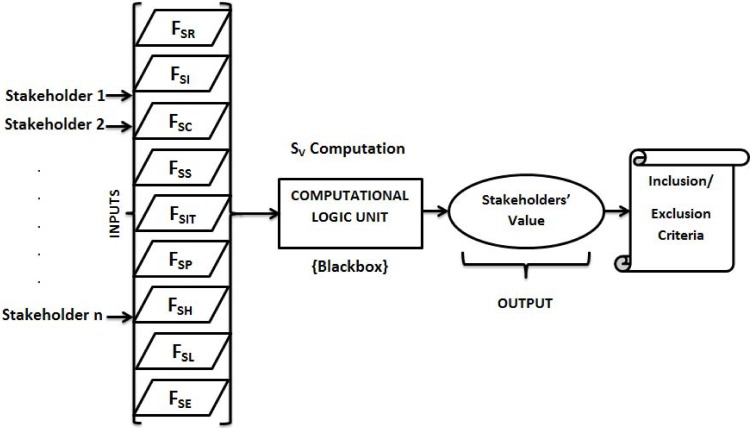
Computational model of stakeholders’ quantification.

The factors given in Table 2 consist of stakeholder aspects and the output is in the form of number values obtained from mathematical formulations as described in Section 4. The values obtained from all these factors serve as an input to the final computational logic in order to find out the worth or value of a stakeholder, which is denoted by *Sv*, for the VBS development. (Fig. 4) depicts the computational model for stakeholder quantification in which stakeholder factors serve as an input to the Computational Logic Unit (CLU) or Blackbox and returns the value of the intended stakeholder based on the proposed computational logic.

### 3.5 Stakeholder Values

The value of stakeholder *Sv* is calculated by using all the stakeholder factor values as a variable. The values of stakeholder factors are given to the final *Sv* equation and quantified value *Sv* of a stakeholder is obtained. The *Sv* is explained in Section 4. The *Sv* is the result of a function which can be written as:

$$\begin{array}{c} Sv=f\left(x\right)\\ x=\left\{F_{SR},F_{SC},F_{SS},F_{SI},F_{SIT},F_{SP},F_{SH},F_{SLG},F_{SE}\right\} \end{array}$$

The output of *f(x)* results in the *Sv* which is used to quantify the stakeholders in order to know the stakeholder importance for the VBS project. The value of ‘*x*’ is based on the values of stakeholder factors. In mathematical notations, *Sv* is a function of *x*. The different factors are taken into consideration in order to quantify the stakeholders which serve as an input to the function. For the calculation of all these factors, different stakeholder aspects are taken into account. These aspects help in finding out the value of a given factor for a given stakeholder. Based on the values of all these factors, the overall value of a stakeholder is calculated which is denoted by the *Sv*.

### 3.6 Inclusion and Exclusion Criteria

The inclusion and exclusion criteria are based on the quantification value of stakeholders obtained from individual stakeholder factors after computation. The inclusion and exclusion criteria depend on the *Sv*. The *Sv* defines that either the involvement of a stakeholder is essential for the success of the system or not in terms of market leverage. The inclusion and exclusion criteria are described in Section 5.

## Stakeholder Factor Formulation

The stakeholder selection is based on the stakeholder metrics, which are termed as ‘factors’ in this study. The stakeholder factors are proposed after long discussions and meetings with the industry professionals. Each of the factor value is calculated by using stakeholder attributes that are stated as ‘aspects’ in this study. In this stage, the experts are heavily involved, and the significance of the stakeholder aspects is defined for different stakeholder factors. A consensus was made by the experts on the use of the terms factors and aspects instead of metrics and attributes. The values of all the proposed factors are calculated by assigning them the values of stakeholder aspects. Different aspects are used for different factors to evaluate the stakeholders thoroughly in order to know their worth for the VBS project. However, in the existing approaches, the use of few aspects does not help in thorough evaluation of the stakeholders and this limitation results in confusion and biases. Thus, a wide range of aspects is evaluated in order to know the worth of a stakeholder for the VBS project. F denotes the stakeholder factor or metric and T denotes the stakeholder attribute or aspect in this research. The values of the aspects are taken as an input on a Likert scale of 0 to 5.

Factor→F

Aspect→T

### 4.1 Stakeholder Risk Factor (F_SR_)

The aspects that are considered for stakeholder risk factor are communication (T_CM_), interpretation (T_IT_), decision-making (T_DM_), cognitive load (T_CL_), complexity (T_CP_), language barriers (T_LB_), time and geographic differences (T_TG_). The selected aspects represent the effect of a stakeholder on the RE and it may be positive or adverse. The details of the selected aspects are as follows:

1. Communication (T_CM_): The aspect of communication is taken into consideration in order to judge that at which level the stakeholder is able to communicate the functional objectives of the project.
2. Interpretation (T_IT_): The aspect of interpretation helps in evaluation of the descriptions given by a stakeholder related to a particular functional aspect of the system.
3. Decision-making (T_DM_): The stakeholder has a prominent role in decision-making or not.
4. Cognitive Load (T_CL_): At which level the stakeholder is able to bear the memory stress.
5. Complexity (T_CP_): To which extent the stakeholder is able to present the complex needs and decisions in a more elaborative way.
6. Language Barriers (T_LB_): The impact of language of the intended stakeholder.
7. Time & Geographic Differences (T_TG_): The impact of time and geographical differences related to the stakeholder.

The bad communication and interpretation may result in high risk in the form of changes or project failure. Same is the case with other aspects in order to judge the risk factor. Initially, the partial effect of each of the aspect is taken in the form of a value and the cumulative effect of the different aspects is calculated by summing up the partial effects of all the aspects. The summation as shown in Equation 1 also helps in reducing the expert biases due to the fact that it prevents from mere numbers game. The same technique is used in all other proposed factors in order to obtain a linear dataset. In the case of F_SR_, if the aspect value is 0 then it means low risk, otherwise 5 means high risk.

$$\begin{array}{c} F_{SR}=0.2\left(T_{CM}+T_{IT}+T_{DM}+T_{CL}+T_{CP}+T_{LB}+T_{TG}\right)+0.2\\ F_{SR}=0.2\left(\sum_{i=1}^{n}F_{SR_{i}}\right)+0.2 \end{array}$$ (1)

The expert assigns the value to each aspect in the range of 0 to 5. For example if the expert assigns the following values to all seven aspects of F_SR_:

$$F_{SR}=0.2\left(3+2+4+3+1+2+3\right)+0.2$$

Then the value of F_SR_ for a given stakeholder is 3.8. In the same way, the values of all other stakeholder factors are calculated based on the aspect values. In Equation 1, *i* denotes an aspect of F_SR_ and *n* is the total number of aspects. After inserting the aspect values in the range of 0 to 5, the output of F_SR_ is in the range of 0.2 to 7.2.

### 4.2 Stakeholder Instability Factor (F_SI_)

The aspects that are considered for stakeholder instability factor are immune to challenges (T_CH_), workload (T_WL_), and fatigue management (T_FM_). These aspects are described as follows:

1. Immune to Challenges (T_CH_): The stakeholder has the ability to face the new challenges.
2. Work Load (T_WL_): The stakeholder is able to bear the extra workload.
3. Fatigue Management (T_FM_): The stakeholder’s expression of fatigue.

In the case of F_SI_, if the aspect value is 0 then it means low risk, otherwise 5 means high risk.

$$\begin{array}{c} F_{SI}=0.2\;\left(T_{CH}+T_{WL}+T_{FM}\right)+0.2\\ F_{SI}=0.2\;\left(\sum_{i=1}^{n}F_{SI_{i}}\right)+0.2 \end{array}$$ (2)

In Equation 2, *i* denotes an aspect of F_SI_ and *n* is the total number of aspects. After inserting the aspect values in the range of 0 to 5, the output of F_SI_ is in the range of 0.2 to 3.2.

### 4.3 Stakeholder Communication Factor (F_SC_)

The aspects that are considered for stakeholder communication factor are clarity (T_CR_), objectivity (T_OB_), and self-confidence (T_SC_). The description of the aspects is given as follows:

1. Clarity (T_CR_): The stakeholder is able to clearly describe the intended needs of the VBS system.
2. Objectivity (T_OB_): The stakeholder is able to properly describe the intended meaning of the required needs in terms of economic benefit.
3. Self-confidence (T_SC_): The level of self-confidence of the stakeholder.

In the case of F_SC_, if the aspect value is 0 then it means that the stakeholder is non-critical, otherwise 5 means critical.

$$\begin{array}{c} F_{SC}=0.2\;(T_{CR}+T_{OB}+T_{SC})+0.2\\ F_{SC}=0.2\;\left(\sum_{i=1}^{n}F_{SC_{i}}\right)+0.2 \end{array}$$ (3)

In Equation 3, *i* denotes an aspect of F_SC_ and *n* is the total number of aspects. After inserting the aspect values in the range of 0 to 5, the output of F_SC_ is in the range of 0.2 to 3.2.

### 4.4 Stakeholder Skill Factor (F_SS_)

The aspects that are considered for stakeholder skill factor are experience (T_EX_), managerial abilities (T_MA_), domain knowledge (T_DK_), domain training (T_DT_), and self-esteem (T_SE_). These aspects are described as follows:

1. Experience (T_EX_): The stakeholder’s prior experience or related experience in the domain of VBS systems.
2. Managerial Abilities (T_MA_): The stakeholder’s level of management in their respective professional area.
3. Domain Knowledge (T_DK_): The current level of domain knowledge of the stakeholder.
4. Domain Training (T_DT_): The stakeholder is properly trained or not.
5. Self-esteem (T_SE_): The stakeholder is holding the status as per his or her required skills.

In the case of F_SS_, if the aspect value is 0 then it means that the stakeholder is non-critical, otherwise 5 means critical.

$$\begin{array}{c} F_{SS}=0.2\;\left(T_{EX}+T_{MA}+T_{DK}+T_{DT}+T_{SE}\right)+0.2\\ F_{SS}=0.2\;\left(\sum_{i=1}^{n}F_{SS_{i}}\right)+0.2 \end{array}$$ (4)

In Equation 4, *i* denotes an aspect of F_SS_ and *n* is the total number of aspects. After inserting the aspect values in the range of 0 to 5, the output of F_SS_ is in the range of 0.2 to 5.2.

### 4.5 Stakeholder Interest Factor (F_SIT_)

The aspects that are considered for stakeholder interest factor are domain scope knowledge (T_DSK_), business knowledge (T_BK_), and objectivity (T_OB_). These aspects are described as follows:

1. Domain Scope Knowledge (T_DSK_): The stakeholder has the knowledge of most relevant subject matter of the domain.
2. Business Knowledge (T_BK_): The stakeholder knows well about the business domain.
3. Objectivity (T_OB_): The knowledge shared by the stakeholder is meaningful in terms of financial value.

In the case of F_SIT_, if the aspect value is 0 then it means that the stakeholder is non-critical, otherwise 5 means critical.

$$\begin{array}{c} F_{SIT}=0.2\;\left(T_{DSK}+T_{BK}+T_{OB}\right)+0.2\\ F_{SIT}=0.2\;\left(\sum_{i=1}^{n}F_{SIT_{i}}\right)+0.2 \end{array}$$ (5)

In Equation 5, *i* denotes an aspect of F_SIT_ and *n* is the total number of aspects. After inserting the aspect values in the range of 0 to 5, the output of F_SIT_ is in the range of 0.2 to 3.2.

### 4.6 Stakeholder Personality Factor (F_SP_)

The aspects that are considered for stakeholder personality factor are cooperative (T_CO_), visionary (T_VI_), inspirer (T_IN_), performer (T_PR_), knowledge sharer (T_KS_), role model (T_RM_), and influence (T_INF_). These aspects are described as follows:

1. Cooperative (T_CO_): The extent of cooperation of a stakeholder with other team members.
2. Visionary (T_VI_): The stakeholder’s level of deep insight of the business.
3. Inspirer (T_IN_): The stakeholder has the ability to do something creative.
4. Performer (T_PR_): The stakeholder has the ability to do something as an achiever in order to make something successful.
5. Knowledge Sharer (T_KS_): The stakeholder shares ideas and experiences with others.
6. Role Model (T_RM_): The stakeholder’s abilities make him or her prominent among team members and other stakeholders consider him or her as a role model.
7. Influence (T_INF_): The stakeholder’s effect on other members in terms of creative thinking, development and behavioral aspects.

In the case of F_SP_, if the aspect value is 0 then it means that the stakeholder is non-critical, otherwise five means critical.

$$\begin{array}{c} F_{SP}=0.2\;\left(T_{CO}+T_{VI}+T_{IN}+T_{PR}+T_{KS}+T_{RM}+T_{INF}\right)+0.2\\ F_{SP}=0.2\;\left(\sum_{i=1}^{n}F_{SP_{i}}\right)+0.2 \end{array}$$ (6)

In Equation 6, *i* denotes an aspect of F_SP_ and *n* is the total number of aspects. After inserting the aspect values in the range of 0 to 5, the output of F_SP_ is in the range of 0.2 to 7.2.

### 4.7 Stakeholder Hierarchy Factor (F_SH_)

The aspects that are considered for stakeholder hierarchy factor are executive position (T_EP_), mid-career (T_MC_), and entry-career (T_EC_). The stakeholder’s hierarchy is rated in terms of high, average and low. The details of these aspects are given as follows.

1. Executive (T_EP_): The stakeholder’s highest level of power based on experience.
2. Mid-Career (T_MC_): The stakeholder’s power level with good experience.
3. Entry Career (T_EC_): The stakeholder is serving as a new team member.

The value of F_SH_ is considered based on the current position of the stakeholders.

$$\begin{array}{l} T_{EP}=\text{High}=4\\ T_{MC}=\text{Average}=3\\ T_{EC}=\text{Low}=2\\ F_{SH}=T_{Val}\\ T_{Val}=Value\;of\;Hierarcy\;Aspects \end{array}$$

### 4.8 Stakeholder Legitimacy Factor (F_SLG_)

The legitimacy shows that either the stakeholder is a legitimate one in terms of system needs or not. The intensity of legitimacy is described in terms of high, average, and low.

$$\begin{array}{l} \text{High}=4\\ \text{Average}=3\\ \text{Low}=2\\ F_{SLG}=T_{Val}\\ T_{Val}=\text{Intensity of Legitimacy} \end{array}$$

### 4.9 Stakeholder Environment Factor (FSE)

The aspects that are considered for stakeholder environment factor are cognitive load (T_CL_), fatigue management (T_FM_), inspirer (T_IN_), and knowledge sharer (T_KS_). There are some of the aspects which are also common in other factors. The commonality exists due to the dual role of these aspects under different scenarios. The detail of these aspects is given as follows:

1. Cognitive Load (T_CL_): At which level the stakeholder is able to bear the memory stress.
2. Fatigue Management (T_FM_): The stakeholder’s expression of fatigue during work hours.
3. Inspirer (T_IN_): The stakeholder has the ability to do something creative.
4. Knowledge Sharer (T_KS_): The stakeholder shares ideas and experiences with others.

In the case of F_SE_, if the aspect value is 0 then it means that the stakeholder is non-critical, otherwise 5 means critical.

$$\begin{array}{c} F_{SE}=0.2\;\left(T_{CL}+T_{FM}+T_{IN}+T_{KS}\right)+0.2\\ F_{SE}=0.2\;\left(\sum_{i=1}^{n}F_{SE_{i}}\right)+0.2 \end{array}$$ (7)

In Equation 7, *i* denotes an aspect of F_SE_ and *n* is the total number of aspects. After inserting the aspects’ values in the range of 0 to 5, the output of F_SE_ is in the range of 0.2 to 4.2.

In all factors, the sum of all different aspects is taken into account and is multiplied by a weight factor of 0.2 and then a weight factor of 0.2 is added to the equation. The summation of the values of all factors is taken into account in order to calculate the accumulative effect of all aspects on the dependant variables of stakeholder factor values. Hence, each factor value is calculated as a linear combination or summation of all the aspects. The weight factor 0.2 is applied in order to get a smaller range of value and to get a linear solution for each factor. The weight factors, greater than 0.2, are problematic in terms of higher values of the *Sv*. Moreover, the weight factor 0.2 is used to standardize the data in a manageable range with upper and lower bounds. The weight factors lower than 0.2 are problematic in terms of fuzzification. Fuzzification causes the problem of uncertainty in the selection process of the stakeholders. Different weight factors are applied and evaluated in the range of 0.1 to 0.9 and it is found that if a factor is applied in this range, the number of quantification values remain the same. However, as the value of the factor is going to increase from 0.1 to 0.9 the values of stakeholder factors are also going to increase. The higher values of stakeholder factors are difficult to manage. Thus, the weight factor of 0.2 is chosen as a solution in order to obtain the normalized and manageable data values. The *Sv* range increases due to the application of a large number of stakeholder aspects. The final *Sv* of a stakeholder is calculated by using the following formula.

$$\begin{array}{c} S_{V}=\left(F_{SC}+F_{SS}+F_{SIT}+F_{SP}+F_{SH}+F_{SLG}+F_{SE}\right)-\left(F_{SR}+F_{SI}\right)\\ S_{V}=\sum_{i=1}^{n}\beta_{i}-\sum_{j=1}^{m}\gamma_{j} \end{array}$$ (8)

$$\beta =\left\{F_{SC}+F_{SS}+F_{SIT}+F_{SP}+F_{SH}+F_{SLG}+F_{SE}\right\}$$ (9)

$$\gamma =\left\{F_{SR}+F_{SI}\right\}$$ (10)

In Equation 8, *β* refers to the values of stakeholder factors that are used to calculate the positive impact of a stakeholder on the system and *i* refers to the *β* factors, where *n* is the total number of *β* factors. However, γ refers to the values of stakeholder factors that are used to calculate the negative impact of a stakeholder on the system and *j* refers to the γ factors, where *m* is the total number of γ factors. After inserting the factor values, the output of *β* is in the range of 5.0 to 31.0 with a geometric progression of 0.2. After inserting the factor values, the output of γ is in the range of 0.4 to 10.4 with a geometric progression of 0.2. *Sv* is the value of stakeholder that is calculated after taking a summation of the *β* values of the factors and negating the summation of the γ values of the factors. The *Sv* is in the range of-5.4 to 30.6 with a geometric progression of 0.2. Equation 9 and Equation 10 shows the *β* and γ factors.

## Inclusion and Exclusion Criterion

The inclusion and exclusion of a stakeholder in the RE process depends on the *Sv*. In case of the *Sv*, the two exceptions are very prominent:

1. The values of *β* and γ cannot be high at the same time.
2. The values of *β* and γ cannot be low at the same time.

These two exceptions show the contradiction of the results. If values of *β* and γ are high at the same time, it means that the stakeholder is highly valuable and highly risky, thus it is not acceptable. On the other hand, if the values of *β* and γ are low at the same time, it means that the stakeholder has low positive value and low risk or negative value, thus it is also not acceptable. Moreover, the requirements engineer may define their own criteria based on the *Sv* of a stakeholder. This makes the proposed framework flexible in terms of stakeholder selection based on the *Sv*.

## Implementation Guidelines

In order to implement the *StakeMeter* framework, the implementers must adhere to some rules and regulations. In this section, some of the key application principles are given in order to implement the proposed framework *StakeMeter*. The application principles are stated as follows:

1. In order to achieve the optimum results of the *StakeMeter* a team of professionals, comprising at least 4 members, must be selected.
2. Get knowledge of the business practices of the organization early for which the product is going to be developed.
3. Apply all steps of the *StakeMeter* in a rigorous way
4. The team members must avoid conflicts and measure the value of each stakeholder based on the proposed framework *StakeMeter*.
5. The team members may divide the organizational units based on their key expertise in order to implement the framework *StakeMeter*.

## Case Studies

Three teams, comprising five members each, are made in order to implement the framework as per given implementation guidelines which are given in Section 6. The purpose to choose the three teams is to evaluate the framework in a true sense and to reduce the extent of biasness. Two members of each team were working on analysis of the stakeholders based on the proposed SIQ framework and on the requirements in order to get the requirements from the intended stakeholders. Two members of the teams were working as developers in order to realize the requirements of the stakeholders into a working system and one member of each team was working on documentation of the system. The teams are made in order to get the unbiased results and to analyse the real worth of the proposed SIQ framework. The description of the projects is given in Table 3.

**Table 3:** Case study descriptions.

Case Study	Acronym	Description
Online Car Show Room	OCSR	An OCSR system is used to sell and purchase new and old cars and also related to the maintenance of the vehicles. The owner possesses a dealership contract with manufacturer. The system is a VBS system in terms of cost benefit.
Hospital Management System	HMS	The system provides better process to manage the day to day activities of the hospital and to provide better medical services to patients. The system is helpful in financial management and is termed as a VBS system.
Restaurant Management System	RMS	The RMS is a business oriented system that is normally used to provide better services to its customers. The system is normally used to keep track of all the transactions that are related to food sales and room bookings by the customers.

The process is initiated by the teams after taking into consideration the total number of stakeholders who are related to the system directly or indirectly. The teams initially met with the top management of the business organizations in order to find out the total number of stakeholders in the given organization. Table 4 lists all the possible stakeholders of an organization.

**Table 4:** Total number of stakeholders.

Organization Name	OCSR	HMS	RMS
Total Stakeholders	23	63	121
Assigned To	Team 1	Team 2	Team 3

### 7.1 Number of stakeholders

In this section, the number of stakeholders of three case studies is described in detail.

#### 7.1.1 Case study: 1 Online Car Show Room

The main purpose of an Online Car Show Room (OCSR) management system is to sell and purchase cars or vehicles, and is normally used by the vehicle dealers. Team 1 who was working on the OCSR found a total of 23 main stakeholders at higher level of abstraction. The car showroom team is presided by one director and two deputy directors. The four stakeholders work on the front desk in order to oversee the customer requests and out of these four, one member works as a cashier. There is one front desk manager who controls the front desk activities. The administration team of car showroom consists of three admin assistants, two accountants and one admin officer. The technical store consists of one store officer, two store assistants, one clerk and five maintenance in-charges. There are 13 mechanics working under these maintenance in-charges who are not directly affected by the system thus, they are not included in the list of stakeholders. Table 5 lists all the stakeholders in the OCSR.

**Table 5:** List of the OCSR stakeholders.

OCSR CASE STUDY		
ID #	Stakeholders	Total
1.	Director	1
2.	Deputy Director	2
3.	Admin Officer	1
4.	Front Desk Manager	1
5.	Store Officer	1
6.	Store Assistant	2
7.	Admin Assistant	3
8.	Accountant	2
9.	Front Desk Employee	4
10.	Clerk	1
11.	Maintenance In-charge	5

#### 7.1.2 Case study 2: Hospital Management System

The Hospital Management System (HMS) is used in order to manage the day-to-day activities of a hospital like administrative affairs, patient administration, billing, and other related activities. The helpful properties of the system are used by all stakeholders from higher ranks to lower ranks in a hospital. Patient is considered as one of the most important entity in this system. There is the need to incorporate all critical needs or requirements of the stakeholders in the intended system. In order to make the HMS beneficial for the intended community, the system should be flexible, reliable, and easy to adopt. The main flow of the information totally depends upon the requirements gathered from the stakeholders. For an automated HMS, there is the need to identify the key stakeholders of the system in order to gather requirements. In the HMS, there is a long list of stakeholders who control different activities of the hospital. The proposed SIQ framework is applied and the stakeholders are quantified based on the *Sv*.

Team 2 was working on the HMS and found a total of 63 stakeholders at a higher level of abstraction. There was one director of the hospital and one administrative officer. The medical departments are governed by four head of departments. In the front desk team, there are five employees headed by a front desk officer. There are a total of 13 medical officers who work in the hospital. The medical laboratory is comprised of three technicians with one lab officer and the X-ray department has two technicians with one X-Ray medical officer. There are a total of 18 male and female nurses. There are two medical equipment maintenance in-charges and three technicians. The admin team consists of one cashier, one accountant, two admin assistants and one clerk. Medical store is controlled by one medical store in-charge and two salesmen. There is one technical store officer and two store assistants. Table 6 lists all the stakeholders in the HMS.

**Table 6:** List of the HMS stakeholders.

HMS CASE STUDY		
ID #	Stakeholders	Total
1.	Director	1
2.	Admin Officer	1
3.	Head of Departments	4
4.	Medical Officers	13
5.	Medical Lab Officer	1
6.	X-Ray Lab Officer	1
7.	Technical Store Officer	1
8.	Nurses	18
9.	Front Desk Officer	1
10.	Front Desk Employee	5
11.	Maintenance In-charge	2
12.	Maintenance Technician	3
13.	Medical Lab Technician	3
14.	X-Ray Technician	2
15.	Technical Store Assistant	2
16.	Medical Store In-charge	1
17.	Accountant	1
18.	Cashier	1
19.	Admin Assistant	1
20.	Clerk	1

#### 7.1.3 Case study 3: Restaurant Management System

The Restaurant Management System (RMS) is assigned to Team 3 and they have listed a total of 121 stakeholders who are working at the restaurant. However, only 21 stakeholders are selected out of 121 and are listed here based on the core responsibilities of the stakeholders. The focused restaurant is supervised by a general manager who is responsible for overall activities of the restaurant. The food manager manages the food supply chains, and the housekeeping manager keeps the maintenance of the building. There is one administrative officer who handles the administrative affairs of the restaurant, two admin assistants and two clerks are working under his supervision. The financial matters are looked by one cashier and one accountant who supervise two assistants. Three supervisors are responsible for the in-house maintenance or needs of the restaurant that should be informed to senior management. The front desk team consists of three employees who guide the guests about room booking and other services and are supervised by one front desk officer. There is one store officer and one store-assistant. Table 7 describes the total stakeholders in the RMS.

**Table 7:** List of the RMS stakeholders.

RMS CASE STUDY		
ID #	Stakeholders	Total
1.	General Manager	1
2.	Food Manager	1
3.	Housekeeping Manager	1
4.	Admin Officer	1
5.	Supervisors	3
6.	Store Officer	1
7.	Store Assistant	1
8.	Accountant	1
9.	Account Assistant	2
10.	Front Desk Executive	1
11.	Front Desk Employee	3
12.	Cashier	1
13.	Admin Assistant	2
14.	Clerk	2

### 7.2 Stakeholders’ responsibilities and grouping

The responsibilities of the stakeholders play a vital role in description of the different business activities. After counting the total number of stakeholders of all three case studies namely OCSR, HMS and RMS, all of the teams asked the lists of responsibilities of all the stakeholders from the respective business organization. Based on these responsibilities, the teams have found dependencies of different stakeholders on each other and the working relationships between all the entities. The teams have divided the stakeholders into different groups based on their responsibilities, especially based on similar responsibilities.

#### 7.2.1 Online car show room

**Fig 5:**
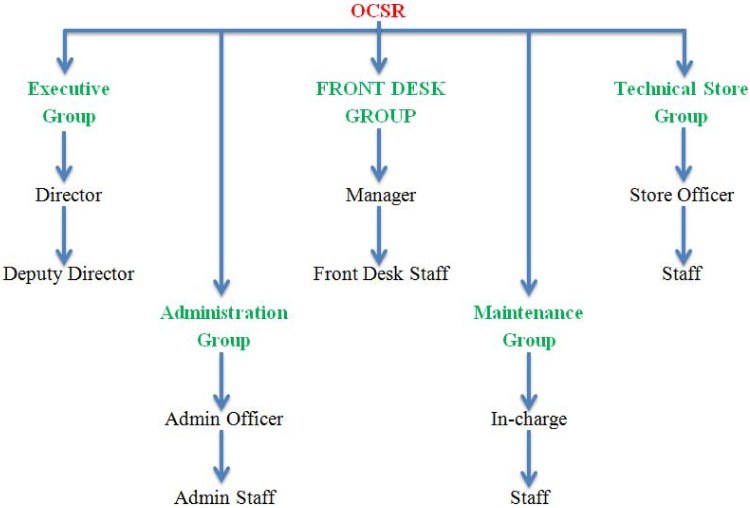
Stakeholders’ groups in OCSR.

Team 1 has divided the stakeholders of the OCSR into five main categories namely executive group, administration group, front desk group, maintenance group and technical store group. These groups help in finding out the hierarchies of the stakeholders in terms of their relative position in the organization. Team 1 has included the director and deputy directors into the executive group who mainly handle the financial issues and overall infrastructure of the showroom. The administration group deals with the administrative affairs of the showroom, thus the administrative officer and staff come in the boundaries of this group. The front desk group comprises front desk officer or manager and the related staff who handle similar activities in the showroom. The maintenance group is related to the maintenance of the old and new vehicles and comprises maintenance in-charges and technicians. The technical store group consists of the store officer and working staff. The technical store group handles the inventory of spare parts in the store of the showroom. (Fig. 5) describes the different groups in the OCSR case study.

#### 7.2.2 Hospital management system

**Fig 6:**
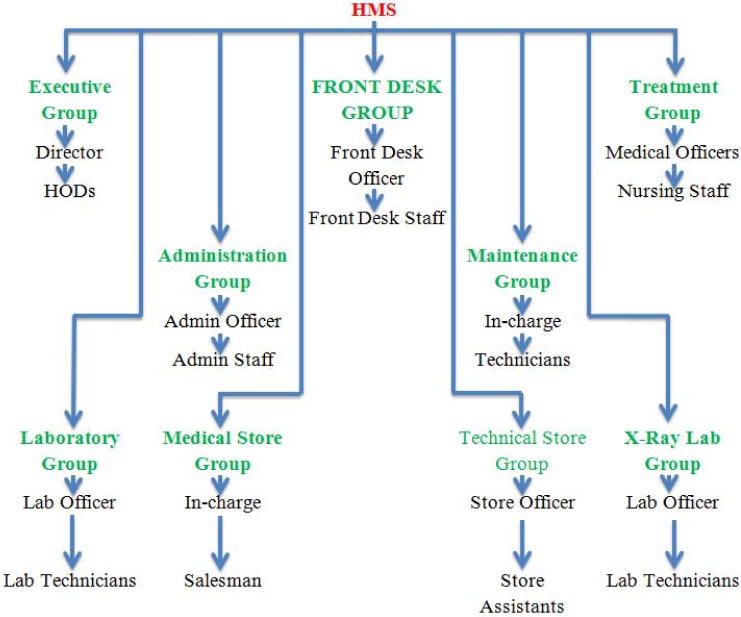
Stakeholders’ groups in HMS.

The team who worked on the HMS divided the stakeholders into nine major groups. The list of groups is comprised of executive group, front desk group, treatment group, administration group, maintenance group, laboratory group, medical store group, technical store group and x-ray lab group. (Fig. 6) lists all the groups of the HMS stakeholders. Executive group consists of director and head of departments. Front desk group contains the list of front desk officer and front desk staff. The treatment group is related to the treatment of the patients and is comprised of medical officers and nurses. The elements of administration group are admin officer and admin staff. Maintenance group contains in-charge and technicians. Laboratory group is related to pathological tests and consists of lab officer and technicians. X-ray laboratory group consists of lab officer and technicians. The medical store group contains store in-charge and salesman. Finally, the technical store group is a set of store officer and store assistants.

#### 7.2.3 Restaurant management system

**Fig 7:**
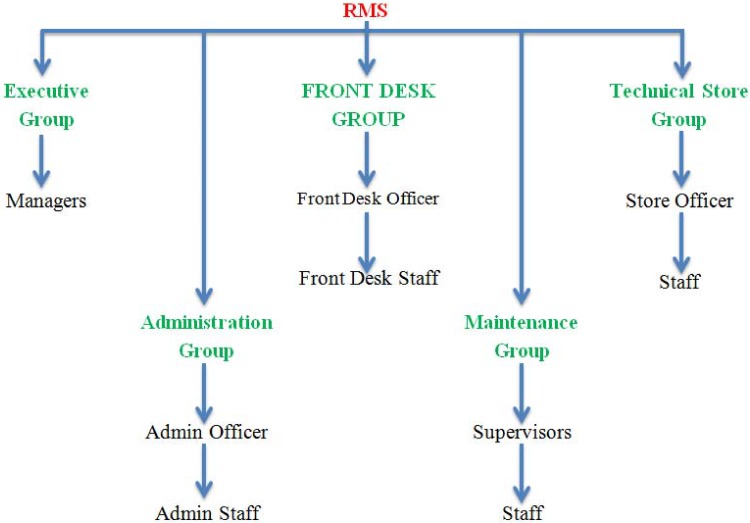
Stakeholders’ groups in RMS.

The software team who worked on the RMS reported five groups of stakeholders, namely executive group, front desk group, technical store group, administration group and maintenance group. (Fig. 7) describes all the possible groups of the stakeholders in the RMS. Executive group contains Managers. Front desk group consists of front desk officer and staff. The technical store group consists of store officer and staff. The administration group consists of admin officer and related staff. The maintenance group is comprised of supervisors and staff.

### 7.3 Stakeholder quantification results and analysis

The stakeholder quantification is performed by applying the proposed factors. Every team has used the proposed nine factors and quantified the stakeholders based on the key attributes. The value of each stakeholder is calculated by using the *Sv* equation. Later, for each project a critical set of stakeholders is selected by the professionals. Table 8 describes the selection and quantification of the OCSR stakeholders based on the proposed factor values and *Sv*. Team 1 of the OCSR selected 13 stakeholders based on the *Sv* of each stakeholder. The *Sv* decreases quickly in the case of the OCSR project, which shows that only few stakeholders are beneficial for this project. The rest of the stakeholders in the case of the OCSR are not included due to the low *Sv*.

**Table 8:** OCSR stakeholder Sv.

Sr. No:	Stakeholder	Group	*β* Values	*γ* Values	S_V_
1.	Director	Executive	26.4	0.8	25.6
2.	Dy. Director	Executive	23.0	1.2	21.8
3.	Front Desk Manager	Front Desk	20.6	1.0	19.6
4.	AO	Administration	18.8	0.8	18.0
5.	SO	TechStore	19.2	1.6	17.6
6.	SA1	TechStore	18.2	1.2	17.0
7.	SA2	TechStore	17.4	0.8	16.6
8.	MI1	Maintenance	17.6	0.8	16.8
9.	MI2	Maintenance	18.8	2.4	16.4
10.	MECH1	Maintenance	16.4	2.0	14.4
11.	MECH2	Maintenance	14.6	1.8	12.8
12.	MECH3	Maintenance	15.2	2.6	12.6
13.	MECH4	Maintenance	12.8	3.2	9.6

Table 9 describes the *Sv* value of the HMS stakeholders. The team has selected 32 stakeholders out of 63 based on the *Sv*. The software development team 2 has considered all those executive stakeholders who have *Sv* greater than 19. The other non-executive stakeholders with *Sv* greater than 15 are included in the set of key stakeholders. The remaining stakeholders are not considered as key stakeholders for the HMS project, and they do not have legitimate needs. The requirements should be gathered from the selected key stakeholders.

**Table 9:** HMS stakeholders’ Sv.

Sr. No:	Stakeholder	Group	*β*	*γ*	S_V_
1.	Director	Executive	24.4	0.6	23.8
2.	Admin Officer	Administration	22.2	0.4	21.8
3.	HOD 1	Executive	25.2	0.8	24.4
4.	HOD 2	Executive	21.6	0.6	21.0
5.	HOD 3	Executive	20.4	0.4	20.0
6.	HOD 4	Executive	22.8	0.6	22.2
7.	FDO	Front Desk	19.2	0.8	18.4
8.	Front Desk Employee 1	Front Desk	20.2	2.2	18.0
9.	Front Desk Employee 2	Front Desk	18.6	1.2	17.4
10.	MO 1	Treatment	21.0	0.8	20.2
11.	MO 2	Treatment	22.4	0.6	21.8
12.	MO 3	Treatment	23.0	0.4	22.6
13.	MO 4	Treatment	20.8	0.6	20.2
14.	MO 5	Treatment	19.8	1.2	18.6
15.	MO 6	Treatment	21.4	0.8	20.6
16.	Lab Officer	Laboratory	18.2	1.0	17.2
17.	Lab Technician	Laboratory	18.4	0.6	17.8
18.	X-Ray Technician	X-Ray Lab	21.2	1.6	19.6
19.	X-Ray MO	X-Ray Lab	17.8	1.2	16.6
20.	Nurse 1	Treatment	19.2	1.2	18.0
21.	Nurse 2	Treatment	22.4	0.6	21.8
22.	Nurse 3	Treatment	21.0	1.2	19.8
23.	Nurse 4	Treatment	22.8	2.2	20.6
24.	Nurse 5	Treatment	18.8	1.6	17.2
25.	Nurse 6	Treatment	23.2	0.4	22.8
26.	Nurse 7	Treatment	17.8	2.4	15.4
27.	Cashier	Admin	21.2	1.0	20.2
28.	Accountant	Admin	22.6	2.4	20.2
29.	Admin Assistant	Admin	19.6	2.6	17.0
30.	Medical Store In-charge	Medical Store	17.8	1.6	16.2
31.	Technical Store Officer	Technical Store	18.0	0.6	17.4
32.	Store Assistant	Technical Store	19.2	1.0	18.2

The description of the *Sv* of the selected stakeholders for the RMS is given in Table 10. Team 3 has selected 21 stakeholders out of 121. The remaining stakeholders are reported as non-critical stakeholders based on the different key aspects as given in the *StakeMeter* framework.

**Table 10:** RMS stakeholder Sv.

Sr. No:	Stakeholder	Group	*β* Values	*γ* Values	S_V_
1.	General Manager	Executive	20.6	1.8	18.8
2.	Food Manager	Executive	22.4	1.6	20.8
3.	Housekeeping Manager	Executive	23.6	1.0	22.6
4.	Admin Officer	Administration	19.8	2.2	17.6
5.	Store Officer	TechStore	18.2	2.0	16.2
6.	Supervisor 1	Maintenance	18.0	2.4	15.6
7.	Supervisor 2	Maintenance	17.8	1.8	16.0
8.	Supervisor 3	Maintenance	19.2	2.8	16.4
9.	Accountant	Administration	18.4	1.2	17.2
10.	Store Assistant	Maintenance	17.6	1.6	16.0
11.	Account Assistant 1	Administration	19.8	2.4	17.4
12.	Account Assistant 2	Administration	18.4	2.0	16.4
13.	Admin Assistant 1	Administration	16.6	2.4	14.2
14.	Admin Assistant 2	Administration	15.2	2.0	13.2
15.	Front Desk Executive	Front Desk	19.8	2.6	17.2
16.	Front Desk Employee 1	Front Desk	15.6	1.8	13.8
17.	Front Desk Employee 2	Front Desk	16.2	2.0	14.2
18.	Front Desk Employee 3	Front Desk	15.4	2.4	13.0
19.	Cashier	Administration	17.8	2.6	15.2
20.	Clerk 1	Administration	16.2	3.0	13.2
21.	Clerk 2	Administration	17.0	2.2	14.8

Software project team 3 who worked on the RMS has selected a total of 21 stakeholders based on the *Sv* of the stakeholders. The trend, which is observed here, is the similarity in the γ values of the stakeholders at all levels, to some extent, and this makes most of the stakeholders significant.

In all of the three case studies, the stakeholders with higher *Sv* are included in the critical set. The *Sv* of each key stakeholder is obtained by using the key attributes of the stakeholder factors. The consultation with these stakeholders proved highly beneficial. The clarity of the ideas of the stakeholders is appreciated by the development team. The emphasis is given on the participation of the highly critical stakeholders in the case of value-driven software systems. Previously, the existing stakeholder analysis approaches are unable to provide a framework that may be adopted for the VBS systems. The empirical findings report that the proposed stakeholder analysis framework has a deeper effect on the success of the VBS system. It is verified by the results that the proposed stakeholder analysis framework is superior in terms of proposed steps, activities and proposed metrics as reported by the team members. The results show that the quantification of key stakeholders depends on the stakeholder aspects or attributes. The existing approaches do not cover the key aspects or attributes in a uniform way due to which the software professionals face different barriers. These barriers make the initiation process obscure. However, the proposed framework makes the initiation of the SIQ easy. During the implementation of the proposed framework, the professionals accepted that the proposed framework focuses on the stakeholders in a vigorous way. The proposed *StakeMeter* framework reduces the burden of software professionals by providing a step by step guide for stakeholder analysis. Hence, the key contribution of the *StakeMeter* framework is based on clear guidelines, adequacy of the attributes, proposed metrics, and implementation details. However, the existing SIQ approaches do not provide support in terms of clear guidelines, attributes and stakeholder metrics. A comparative analysis of the *StakeMeter* framework, based on the key contributions, is shown in Section 8 with respect to the existing SIQ approaches and methods.

## Performance Analysis of the StakeMeter

In order to measure the performance of the *StakeMeter* framework three existing methods are selected for comparative analysis. One is very initial method called as Mitchells’ method (1997) and the second is a matured method as compared to the Mitchells’ method and is called as Ballejos & Montagna method [28]. The third method is a latest one and is based on the bi-metric and fuzzy c-means algorithm [36]. The comparative analysis is based on the number of explored stakeholders, time consumption in terms of total man hours for stakeholder analysis, clearly defined priorities and detailed guidelines. The time less than 24 man hours is considered as low, the time greater than 24 and less than or equal to 48 man hours is considered as medium and lastly the time greater than 48 man hours is considered as high. Table 11 shows the comparative analysis of the proposed *StakeMeter* framework with the selected SIQ methods.

**Table 11:** Comparative analysis.

	Stakeholders			Time-consumption			Defined-Priorities of the Stakeholders	Detailed Guidelines
	OCSR	HMS	RMS	OCSR	HMS	RMS		
**Mitchells’ Method**	18	43	39	High	High	High	×	×
**Ballejos & Montagna Method**	15	46	53	Medium	High	High	×	Yes
**Bi-Metric & Fuzzy C-Means Method**	8	22	13	Low	Low	Low	Yes	Partial
**StakeMeter**	13	32	21	Low	Low	Medium	Yes	Yes

Table 11 shows the comparative analysis of the three case studies of the OCSR, HMS, and RMS. Along with the application of *StakeMeter* the teams have applied Mitchells’ method, the method proposed by Ballejos and Montagna and lastly the bi-metric and fuzzy c-means method in order to analyse the stakeholders of the selected case studies. However, the Mitchells’ method is applied first of all. Secondly, the method proposed by Ballejos and Montagna is applied. Thirdly, the bi-metric and fuzzy c-means based method is applied. The main purpose to apply the three methods, prior to the *StakeMeter* application, is to find out the performance of the three methods and latter their performance is compared with the *StakeMeter* framework. Moreover, the teams are assigned every time a new case study in order to avoid expert biases.

### 8.1 Applications of the Mitchells’ method

Initially, in the first step Mitchells’ method is applied. The team working on the OCSR case study has found 18 stakeholders and it is reported that the time-consumption is very high. In the case of Mitchells’ method, it is difficult to define the individual priorities of the stakeholders. Moreover, the guidelines of Mitchells’ method are too abstract. There is a lack of low level details of the activities. The team working on the HMS case study has applied Mitchells’ method and explored 43 stakeholders and the time taken during stakeholder analysis is high. Later, the Mitchells’ method is applied on the RMS study. The team working on the RMS case study has found 39 stakeholders. The time-consumption is also high. The guidelines in the case of Mitchells’ method are not clear and this results in higher time-consumption and large number of stakeholders is explored in each case study. It is observed that most of the stakeholders are treated at par instead of quantifying them individually.

### 8.2 Applications of the Ballejos and Montagna method

Before application of Ballejos and Montagna method [28] all three teams are shuffled on different projects in order to avoid any bias and to measure the performance of the *StakeMeter* effectively. Team working on the OCSR case study has reported 15 stakeholders and the time taken to analyse the stakeholders is medium. The team working on the HMS case study has found 46 stakeholders and the time consumed in the case of HMS case study is high in terms of total man hours. The team of the RMS case study explored 53 stakeholders by applying Ballejos and Montagna method. The time reported by the team in terms of total man hours is high. In the case of Ballejos and Montagna method, it is also observed that more stakeholders are the part of game as compared to the Mitchells’ method. Later, it is analyzed by the experts in each case study which stakeholders are not the key stakeholders and all these stakeholders are not considered as vital for the VBS development.

### 8.3 Application of the bi-metric and fuzzy c-means method

The bi-metric and fuzzy c-means based stakeholder analysis method is a most recent method. The details of the application of this method are given in [36]. The quantification of the stakeholders is based on two stakeholder metrics named as stakeholder skill factor and stakeholder interest factor. It is reported by the professionals that the given skill and interest factors result in selection of only those stakeholders who possess an executive role in the community and many other stakeholders are neglected. The two metrics mainly focus on the domain and its knowledge. However, the requirements of the stakeholders may vary under different contexts which make the suitability of the proposed metrics questionable for the projects with the large number of stakeholders. In this method, few stakeholders are selected based on the proposed metrics as shown in Table 11. However, it is also reported that the use of fuzzy c-means method may serve well in dividing the stakeholders into different clusters based on their proposed values. The proposed method is efficient, but some of the stakeholders are missing in this case and are not the part of critical stakeholders. The team working on the OCSR has reported eight key stakeholders. The team working on the HMS case study explored 22 stakeholders as key stakeholders. However, the RMS team explored a total of 13 stakeholders as critical. The bi-metric and fuzzy c-means method neglects the stakeholders due to the focused attributes which mainly help in the selection of executive members only. This problem is solved by the *StakeMeter* framework in which stakeholder factors or metrics are divided into two main categories of *β* and γ factors. The *β* and γ factors cover a wide range of the stakeholder aspects and make the stakeholder analysis process lenient. The proposed factors of the *StakeMeter* help in evaluation of the stakeholders at different levels who are interested in the development of the VBS project. In the case of *StakeMeter* framework, a range of stakeholder factors mitigates the extent of expert biases too.

The bi-metric and fuzzy c-means based method [36] does not provide the low level implementation details in order to initiate the SIQ. The problem of lack of low level details is highlighted in (Fig. 1). The proposed stakeholder quantification framework *StakeMeter* provides a set of guidelines for industry professionals in order to quantify or prioritize the stakeholders. However, the bi-metric and fuzzy c-means based method is unable to provide easy to adopt guidelines for industry professionals. In the *StakeMeter*, the divide and conquer approach is applied in which the stakeholders are grouped into different categories based on their responsibilities. The responsibilities are assessed through job cards. Based on these responsibilities it becomes easy to find out the initial worth of a stakeholder. However, it is not so in the case of bi-metric and fuzzy c-means based method. Later, in *StakeMeter* the entities are evaluated based on the key aspects and factors that are discussed in Section 4. The proposed framework *StakeMeter* adds new knowledge to software engineering body of knowledge (SWEBOK) and provides full support to industry professionals in the SIQ initiation.

### 8.4 Application of the StakeMeter framework

The details of the application of the *StakeMeter* framework are given in Section 7. The team working on the OCSR has explored 13 key stakeholders and the time consumed in this case is low. In the case of HMS case study, the team has reported 32 stakeholders and the reported time is low again. Moreover, in the case of RMS case study the number of reported stakeholders is 21 and the time taken to analyse the stakeholders is medium as compared to the other three methods. The total number of stakeholders explored by the Mitchells’ method, and Ballejos & Montagna method in the case of RMS is high as compared to the *StakeMeter* framework. The *StakeMeter* framework has identified the critical stakeholders only. In the case of bi-metric and fuzzy c-means method, the effort in man hours is less but some of the key stakeholders are missing as compared to the *StakeMeter* framework. However, in the case of the *StakeMeter* framework the overall man hours are less as compared to the Mitchells’ method, and Ballejos & Montagna method.

**Fig 8:**
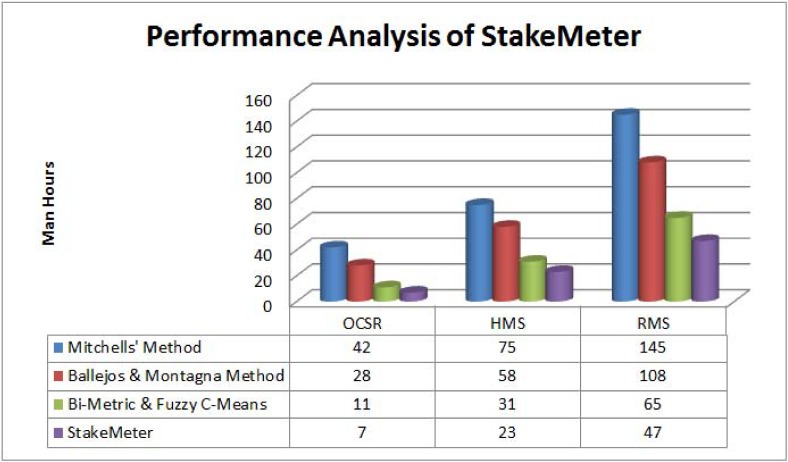
Performance analysis of StakeMeter.

The performance analysis of the three research studies is shown in (Fig. 8). In (Fig. 8), the effectiveness of the three research studies is measured in terms of time and it is obvious from the graph that the efficiency of the proposed *StakeMeter* is higher as compared to the other studies.

The experts of all three teams later analyzed the results and reported their observations. It is observed that due to the lack of clear quantification criteria it is difficult to define the baseline for inclusion and exclusion of stakeholders in the case of Mitchells’ method, Ballejos and Montagna method and bi-metric fuzzy c-means method for the VBS development process. Initially, the results of the *StakeMeter* framework are analyzed by the experts. It is reported that in the case of OCSR one stakeholder MECH4 has high risk and low *Sv* as compared to the other mechanics. Hence, it is decided that it is not an element of the set of success critical stakeholders. At this initial stage this stakeholder is eliminated from the key stakeholder set. In the case of HMS, experts have also eliminated one stakeholder Nurse 7 due to the high risk and low *Sv*. However, in the case of RMS 3 stakeholders were eliminated by the experts in the first scrutiny due to the low *Sv*. Hence, in the final dataset of the stakeholders there are 12 key stakeholders in the OCSR, 31 in the HMS, and 18 in the case of RMS.

In the case of Mitchells’ method, the number of selected stakeholders is high as compared to the *StakeMeter*. As compared to *StakeMeter* six non-key stakeholders are selected with the application of Mitchells’ method in the case of OCSR. In the case of HMS, 12 non-key stakeholders are selected and in the case of RMS 21 non-key stakeholders are selected.

The number of explored stakeholders in the case of Ballejos and Montagna method is also high as compared to the *StakeMeter* framework. In the case of OCSR, three non-key stakeholders are identified as key stakeholders. In the case of HMS, 15 non-key stakeholders are identified as key stakeholders and in the RMS case study 35 non-key stakeholders are reported as success critical stakeholders.

**Fig 9:**
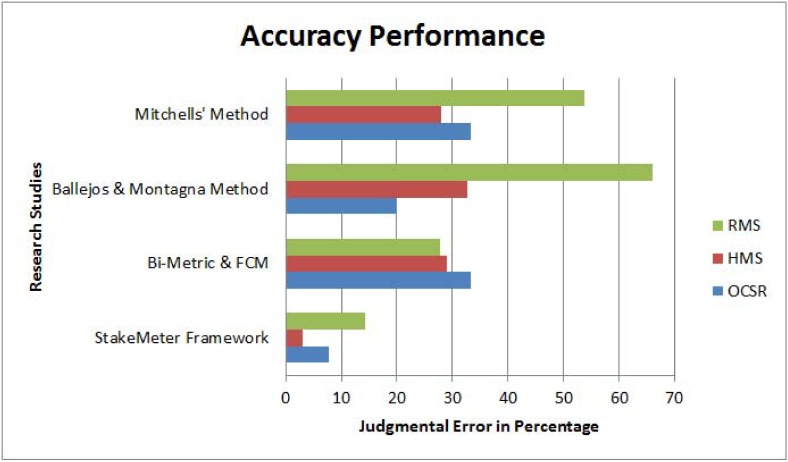
Accuracy analysis of StakeMeter.

In the case of bi-metric and fuzzy c-means method, the number of selected stakeholders is less as compared to the *StakeMeter* framework. In the OCSR case study four key stakeholders are missing. In the case of HMS case study nine key stakeholders are missing while in the case of RMS case study five key stakeholders are missing. This method mainly selects the executive or influential stakeholders of the VBS system and ignores others. Hence, in this method the error is based on the less number of selected stakeholders and some of the key stakeholders are neglected. In this case the error is higher than the *StakeMeter*. The identification error in the case of *StakeMeter* is very low as compared to the two other methods. Based on the three, case studies the judgmental errors of the three research studies are shown in (Fig. 9) in the form of percentages. (Fig. 9) shows that the error in the case of *StakeMeter* framework is very low as compared to the other studies.

Moreover, in order to measure the performance of the proposed SIQ framework *StakeMeter* the industry professionals are also involved for better understanding. Industry professionals have analyzed and evaluated the framework by applying it on different projects. The data is gathered from the industry professionals about performance of the proposed framework *StakeMeter* using survey questionnaire. Two surveys are conducted in this study one before application of *StakeMeter* and one after application of *StakeMeter*. The questionnaire is based on the five key parameters of standardization, easiness, efficiency with respect to time, ambiguity or lack of clarity of the proposed framework. The questionnaire is sent to 25 industry professionals in order to record the data about performance analysis of the proposed framework. The response rate of the different reviewers, before and after application of *StakeMeter*, is shown in Table 12. Previously it is reported by 95.40% of the respondents, that the existing SIQ approaches are not standardized. However, in the current survey, it was reported by 80% of the respondents that the proposed framework *StakeMeter* can be adopted as a framework and 20% of the respondents found that it is still difficult. It was reported by 80% of the respondents in the previous survey that the existing SIQ approaches are not easy to carryout. About the proposed framework *StakeMeter* it is reported by 84% of the respondents that the proposed framework is easy to understand and implement. Moreover, 16% of the respondents reported that the framework is difficult. Previously, it is reported by 72.42% of the respondents that the existing SIQ approaches are difficult to apply in terms of lack of clear guidelines. However, about 88% of the respondents reported that the proposed framework *StakeMeter* provides clear and easy to understand guidelines and 12% are of the view that the framework is not easy. The responses given by most of the respondents show that the proposed framework *StakeMeter* is easy, efficient in terms of time, clear and can be standardized. Table 12 shows the performance of the proposed framework *StakeMeter*.

**Table 12:** Response rate of StakeMeter performance.

Sr. No:	Problem	Before Response	After Response
1	Lack of Standardization	95.40%	20.0%
2	Not Easy	80.0%	16.0%
3	Time-consuming	78.0%	12.0%
4	Ambiguity or lack of clarity	72.42%	12.0%

The comparison of the *StakeMeter* framework is also made with different methods and is shown in Table 13. The key elements that are taken into account in the comparison are personality metrics, technical metrics, inclusion/exclusion criteria, attribute adequacy, complexity, low level descriptions or guidelines and cost effectiveness in terms of time consumption. The proposed framework *StakeMeter* deals with all the key issues of stakeholder analysis. However, there are some methods which provide partial or limited support for the different key parameters that are focused during comparative analysis and most of the key parameters are not focused by the other methods and approaches.

**Table 13:** Comparative Analysis of Different Methods.

Method	PersonalityMetrics	Technical Metrics	Selection Criteria	Attribute Adequacy	Complexity	Guidelines	Cost Effective
**StakeMeter**	Yes	Yes	Yes	Yes	Yes	Yes	Yes
**Ballejos & Montagna [39]**	Partial	×	Partial	×	×	Yes	×
**Fuentes et al. [40]**	×	×	×	×	×	×	×
**Ballejos & Montagna [28]**	×	×	×	×	×	Partial	×
**Press & Wegmann [22]**	×	×	×	×	×	Limited	×
**Babar et al. [36]**	Limited	Limited	Partial	Partial	Yes	Partial	Yes
**Boonstra [30]**	×	×	×	×	×	×	×
**Mitchel et al. [21]**	×	×	×	×	×	Partial	×
**Coakes & Elliman [24]**	×	×	×	×	×	×	×
**Pan [41]**	×	×	×	×	×	×	×
**Pouloudi [42]**	×	×	×	×	×	Limited	×
**Whitley et al. [43]**	×	×	×	×	×	×	×
**McManus [20]**	×	×	×	×	×	Partial	×
**Glinz & Wieringa [31]**	×	×	×	×	×	Partial	×
**Razali & Anwar [44]**	×	×	×	×	×	Partial	×
**Power [45]**	×	×	×	×	×	Partial	×
**Lim et al. [46]**	×	×	×	×	Partial	Yes	Partial

## Future Research Directions

The future research is based on two phases. The first phase is to calculate the trustworthiness of all existing stakeholder analysis approaches in a rigorous way. The second phase of the research is to design an intelligent solution in order to reduce the extent of complexity and expert biases. The proposed framework *StakeMeter* is given for industry professionals. In order to evaluate the trustworthiness of the proposed *StakeMeter*, there is a need to evaluate the trustworthiness of all the existing stakeholder analysis approaches. By finding out the support given by each approach it will be easy to measure the level of expert satisfaction. Hence, in order to measure the satisfaction of the industry professionals or experts in terms of stakeholder analysis approaches we hereby suggest the key satisfaction estimation metrics as proposed in [13].

In the first step, the similarity between the two approaches is calculated by applying Pearson Correlation Coefficient (PCC). Let us suppose, there are N experts and K stakeholder approaches. The expert-approach matrix (EAM) for efficiency (*e*) value prediction is defined as:

$$EAM=\left[\begin{array}{ccc} e_{1,1} & \cdots & e_{1,K}\\ \vdots & \ddots & \vdots \\ e_{N,1} & \cdots & e_{N,K} \end{array}\right]$$

For two approaches *SA*_*i*_ and *SA*_*k*_ the PCC is applied in order to calculate the similarity between the two by using the following formula:

$$\begin{array}{l} Sim\left(SA_{i},SA_{k}\right)=\frac{\sum_{n\in U}\left(e_{n,i}-{\bar{e}}_{i}\right)\left(e_{n,k}-{\bar{e}}_{k}\right)}{\sqrt{\sum_{n\in U}{\left(e_{n,i}-{\bar{e}}_{i}\right)}^{2}}\sqrt{\sum_{n\in U}{\left(e_{n,k}-{\bar{e}}_{k}\right)}^{2}}} \end{array}$$ (11)

The similarity value between the two approaches is used to predict the value of the target approach which is *StakeMeter* in this research. Ding et al. (2013) have later identified customer satisfaction, related to a cloud service, as a linear combination of perception function *f*_*p*_ and disconfirmation function *f*_*d*_ as proposed in the CSAT model [37]. Hence, the same function is suggested here in this research in order to estimate the satisfaction of an industry professional or expert with respect to the efficiency of the existing stakeholder approaches. Let us suppose, for a target stakeholder approach *SA*_*i*_ the expert satisfaction is denoted as:

$$EX_{n}\left(r_{t}\right)=f_{p}\left(r_{t}\right)+f_{d}\left(\frac{r_{t}-r_{t}^{-}}{r_{t}^{+}-r_{t}^{-}}\right)$$ (12)

In order to evaluate the utility of the approach the constant relative risk aversion (CRAA) function can be applied as proposed in [38]. Lastly, the trustworthiness of an approach can be calculated by using the *trust*_*n*_ equation as given in [13].

The next phase for future research is to propose the multi-criteria based neuro-fuzzy inspired intelligent decision support system in order to reduce the extent of complexity and expert biases.

## Conclusion

The existing stakeholder quantification approaches are non-uniform, inconsistent, and time consuming. Moreover, the existing approaches do not provide the low level implementation details and stakeholder quantification metrics or factors. All these SIQ issues serve as a motivation for this research. Hence, this research study contributes in the form of a new SIQ framework called *StakeMeter* based on the stakeholders’ aspects and factors for the VBS systems. The framework is highly beneficial in terms of elaborated activities defined in the process. Moreover, the framework can also be used generically in the industry. The proposed SIQ framework provides professional support in stakeholder analysis and RE to business analysts and developers. The proposed framework *StakeMeter* provides an easy way to initiate it as compared to the other proposed approaches and methods. Due to the unclear guidelines of the existing approaches the time spent on stakeholder analysis spans over several months and years. However, the time taken by the said framework *StakeMeter* is very less as compared to the other SIQ approaches and methods. The proposed stakeholder factors add knowledge to the SWEBOK and also support industry professionals.

However, still the study has some limitations and threats. Firstly, the threat is associated with the validity of the *StakeMeter* if there is a lack of expertise in the stakeholder analysis. The framework requires good expertise in stakeholder analysis domain in order to understand and interpret the requirements. Expert analysts can only evaluate the stakeholders based on the proposed framework and key factors. The professional expertise is highly desirable in order to initiate the framework. Secondly, the framework needs to be implemented in large projects with hundreds of stakeholders and requirements in order to check its performance. So far, the framework is applied on projects with small number of stakeholders. The application in larger projects will help in better evaluation of latency issues in terms of time and other stakeholder management problems. Thirdly, the framework is not tested for the remote stakeholders. There is also the need to apply it in global software engineering practices for better applicability. Lastly, there are chances of the biases induced by the experts which can be reduced by proposing an intelligent solution for the SIQ process.
